# What drives me to use TikTok: A latent profile analysis of users’ motives

**DOI:** 10.3389/fpsyg.2022.992824

**Published:** 2022-12-01

**Authors:** Li Gu, Xun Gao, Yong Li

**Affiliations:** School of Innovation Design, Guangzhou Academy of Fine Arts, Guangzhou, China

**Keywords:** TikTok use, latent profile analysis, motives, uses and gratifications theory, escapist addiction

## Abstract

With TikTok rising in social media, the heterogeneity of users creates diversity in motives for using it. The issue of which profiles of TikTok use motives can be identified warrants greater understanding. Conducting latent profile analyses for a sample of 384 TikTok users, we identified four profiles, namely overall low motives, overall medium motives, overall high motives, and escapist addiction and novelty motives profiles. The former three profiles reflect different levels of motivations across four motives (socially rewarding self-presentation, trendiness, escapist addiction, and novelty). It is worth noting that TikTok users in the escapist addiction and novelty motives profile are mainly motivated by escapist addiction and novelty, but not socially rewarding self-presentation or trendiness motives. Through multivariate analysis of variance (MANOVA) and multinomial logistic regression, we further explore the differences in TikTok use between the profiles and the extent to which users’ background characteristics and TikTok use predict their profile membership. The four profiles differ significantly in terms of the scores of almost all TikTok use motives. The frequency of TikTok use and video posting are the most notable predictors of profile membership. These results make theoretical contributions to the extant literature on social media use profiles by exploring the number and types of latent profiles of TikTok use motives, which also inform opportunities for enhancing user experience and imply tailored content recommendations to both achieve maximized gratifications and maintain mental wellbeing based on user profiles.

## Introduction

Since its debut in 2016, the social media app TikTok (“DouYin”) has drawn a wide variety of users and stands out from the ferocious competition. In terms of both downloads (Briskman, 2022) and monthly use in minutes (Southern, 2021), TikTok has exceeded other well-known social media apps, such as Facebook and Instagram. TikTok is currently a worldwide phenomenon with over 1 billion users and is accessible in over 150 countries. It has been downloaded more than 200 million times only in the United States (Doyle, 2022). In China, the number of active daily users of the app is 600 million (Scherr and Wang, 2021).

Given the prominence of TikTok, studies from different cultures have focused on its use in various contexts, such as entertainment (Meng and Leung, 2021), political communication (Cervi et al., 2021), and infodemiology (Purushothaman et al., 2022). The motivations underlying its use have drawn researchers’ attention (for review, see Montag et al., 2021). Empirical research on the TikTok use motives is mainly about descriptive overviews and variable-centered findings (e.g., Omar and Dequan, 2020; Scherr and Wang, 2021). For instance, a recent study demonstrated that four distinct motivations, including socially rewarding self-presentation, trendiness, escapist addiction, and novelty, motivated Chinese users to utilize the social media app TikTok (Scherr and Wang, 2021). The socially rewarding self-presentation motivation refers to socially rewarding and valued gratification arising from TikTok’s feature of self-generating and uploading content. TikTok users could enhance relationships with friends and family and also make new friends. The trendiness motivation refers to that TikTok impresses users as being fun to use and popular, encouraging people to use it. The escapist addiction motivation for using TikTok taps into cognitive restoration and describes how users use the app to escape from their daily lives and forget about the troubles. The novelty motivation taps into the freshness and originality of the TikTok content, thus motivating people to use it.

Although distinct motives for TikTok use have been proposed, tailed content recommendations for homogeneous subgroups of users based on different patterns of motives should be provided. Since TikTok users are heterogeneous regarding their personal experiences, they are diverse in the combinations of TikTok motives. Content personalization is crucial for users (Zanker et al., 2019), and it is important for TikTok app to provide tailored content recommendations to achieve maximized gratifications. The current study consequently examines how to explore TikTok users’ motive profile in order to provide personalized content recommendations. More specifically, by means of latent profile analysis (LPA), the current study provides an answer to the question of which unobserved (latent) TikTok use motives profiles exist based on the aforementioned four motives (ie., socially rewarding self-presentation, trendiness, escapist addiction, and novelty). To further integrate and account for the diversity of TikTok users, the current study explores whether users’ background traits and TikTok use can predict their profile memberships.

## Theoretical background

### Uses and gratification theory and TikTok use

Uses and gratification theory was developed and used to explain diverse media use practices (Katz and Foulkes, 1962; Katz et al., 1973). This theory highlights that people use mass media to satisfy particular wants and aspirations (Katz, 1959; Katz et al., 1973). Although this theory was created to explain how people use mass media, it is now frequently used to explain how people use social media, such as Facebook (Nadkarni and Hofmann, 2012; Cristescu and Balog, 2018; Kowal et al., 2020; Raza et al., 2020), Tinder (Sumter et al., 2017; Timmermans and De Caluwé, 2017), and Instagram (Lee et al., 2015; Phua et al., 2017).

TikTok’s excellent social media commercial performance has drawn researchers’ attention, such as motivations for using TikTok app. In recent studies, the use of TikTok has been examined using the uses and gratification theory (Bossen and Kottasz, 2020; Omar and Dequan, 2020). Bossen and Kottasz (2020) adopted the uses and gratification theory to better understand TikTok use among pre-teenagers and adolescents. They showed that passive consumption was common and that users’ behavior on TikTok, including passive content consumption as well as participation and contributory activities, was mostly motivated by fulfillment of amusement or affect. Omar and Dequan (2020) discovered that TikTok usage was significantly predicted by archiving, self-expression, social connection, and escapism. Moreover, Scherr and Wang (2021) discovered four main motives why Chinese people use TikTok, explaining the success of TikTok with gratification niches. They also demonstrated the roles of these motives in driving TikTok use behaviors. Specifically, daytime use was driven by trendiness, nighttime use was driven by novelty, and posting TikTok videos was driven by socially rewarding self-presentations. In general, prior studies have proposed underlying motivations for using TikTok based on uses and gratification theory.

### Person-oriented approach to social media use

Descriptive summaries and findings with a focus on variables currently dominate the empirical literature on the reasons people use social media, especially TikTok (e.g., see Best et al., 2014; Bowden-Green et al., 2021; Meng and Leung, 2021). More in-depth analyses, particularly those into interindividual variance, are constrained by these correlational techniques (Jung and Wickrama, 2008). The person-centered technique known as latent profile analysis (LPA) is a significant and relevant addition to the body of knowledge. According to B. Muthén and Muthén (2000), LPA is a cross-sectional methodology that emphasizes interactions between people rather than factors. Based on self-reported response patterns to continuous data, the goal is to categorize individuals into separate meaningful groups (i.e., latent profiles) (Jung and Wickrama, 2008). LPA has been successfully used in many domains to identify subpopulations based on a multivariate set of observed attributes (Caffaro et al., 2017; Vanslambrouck et al., 2019; Spurk et al., 2020).

A few research (Scott et al., 2017; Lo Coco et al., 2018; Shensa et al., 2018) have used person-centered approaches to examine social media user profiles in terms of several metrics. For instance, Scott et al. (2017) developed distinct 3-profile solutions for emerging adults’ social media use in terms of frequency and engagement. For the frequency of social media use, the low frequency profile, the medium frequency profile, and the high frequency profile were derived. Similar three profiles were derived, namely the minimally engaged profile, the moderately engaged profile, and the highly engaged profile. Lo Coco et al. (2018) empirically determined three homogeneous groups of Facebook users based on variables regarding their Facebook usage (e.g., time spent, pictures posted, and number of friends), which were labeled as mild-users, committed to Facebook, and online socially-oriented groups. Moreover, they also found that each subgroup could be characterized by a specific pattern of personality characteristics. These studies focused mainly on Facebook user profiles. To the best of our knowledge, the research question regarding the identification of homogeneous subgroups of TikTok users remains unanswered. TikTok shares commonalities and dissimilarities with other social media platforms such as Facebook and Instagram. Given its rapid rise to prominence, it is worth looking into TikTok user subgroups based on TikTok motives, thus providing tailed content recommendations and enhancing engagement.

### The link between social media use and background characteristics

The links between social media use and background characteristics (e.g., age and gender) are well-documented (McAndrew and Jeong, 2012; Scott et al., 2017; Laor, 2022). For instance, it has been demonstrated that females, younger people, and those not currently in a committed relationship were the most active Facebook users (McAndrew and Jeong, 2012). Additionally, Scott et al. (2017) found that females dominated the group of frequent social media users. Investigating the variations in TikTok use motives based on various user backgrounds is both fascinating and important given the diversity of TikTok users. The majority of TikTok users are young, with over 80% of Chinese users being under 35 years old and the majority of US users being under 30 years old (Montag et al., 2021). It has been shown that age was associated with trendiness and escapist addiction among TikTok use motives, and that there was a gender difference for addictive escapism (Scherr and Wang, 2021). Based on these findings, we predicted that age and gender might play a role in why people utilize TikTok. Furthermore, social media usage metrics such as frequency and engagement were crucial (Scott et al., 2017). We hypothesize that the motivations behind TikTok use are sensitive to TikTok use (e.g., frequency, daily use time, and video posting).

## Present research

Due to the different design and usability features of TikTok compared to other popular SNSs like Facebook, Twitter, and Instagram, users of TikTok may differ significantly in motives for using, and gratifications derived from it. Although previous research has proposed motives for TikTok use, tailed content recommendations for homogeneous subgroups of users should be provided to achieve maximized gratifications. Due to the diversity of their personal experiences, TikTok users are diverse in the combinations of TikTok motives. By means of motive-based latent profile analysis (LPA), the current study explores the number and types of latent profiles among a sample of TikTok users in China. To further integrate and account for the diversity of TikTok users, the current study is also interested in examining if derived profiles could be predicted by demographic and individual characteristics (e.g., age, gender, education, and TikTok use).

## Materials and methods

### Participants

Participants were 384 TikTok users aged 17–58, residing in China, and with experience using TikTok. They were recruited and interviewed by the Chinese survey sample provider Wenjuanxing^1^ in 2022. Wenjuanxing is a reputable survey company in China, with 2.6 million people enrolled on the platform. The questionnaire was fully anonymous, and participants were told that all information collected would only be utilized for academic study. These guidelines enabled participants to respond to questions more frankly and openly. All participants gave their informed consent. Table 1 presents the characteristics of the sample. The average age of participants was 27.74 years (SD = 8.92; range 17–58). Slightly more than half were women (61.98%) and students (51.30%). Most participants (83.59%) reported a monthly income below ¥10,000, and 76.04% reported having at least some college education.

**Table 1:** Characteristics of participants.

Participant characteristics	N (%)
Age	*M* = 27.74 (SD = 8.92)
Gender	
Men	146 (38.02)
Female	238 (61.98)
Student status	
No	187 (48.70)
Yes	197 (51.30)
Education	
High school/GED or less	27 (7.03)
Associate’s degree	65 (16.93)
Bachelor’s degree	221 (57.55)
Master’s degree/Doctorate	71 (18.49)
Monthly Income	
Less than ¥2,000	123 (32.03)
¥2,000–4,999	127 (33.07)
¥5,000–9,999	71 (18.49)
¥10,000–14,999	27 (7.03)
¥15,000–19,999	14 (3.65)
¥20,000 or more	22 (5.73)

Gender was coded: 1 = man; 2 = woman. Student status was coded: 0 = not a student; 1 = currently a student.

### Measures

#### Motives for TikTok use

For motives for TikTok use, we used the measure reported by Scherr and Wang (2021), who developed and validated a self-reported measure of TikTok use motive with 1,051 TikTok users in China. For all items, agreement with the statements was assessed on a Likert-type scale ranging from 1 = totally disagree to 5 = totally agree. The scale consists of four subscales, i.e., Socially Rewarding Self-Presentation, Trendiness, Escapist Addiction, and Novelty (1 item). The reliability is 0.926, 0.849, and 0.884 for the former three subscales, respectively.

#### Tiktok use

##### Daily use tim*e*

We assessed the daily use time of TikTok as an average for each day on a scale ranging from 1 = less than 10 min, 2 = 11–30 min, 3 = 31–60 min, to 4 = more than 1 h.

##### Frequency of TikTok use

We assessed the frequency of TikTok use on a scale ranging from 1 = every day, 2 = once every two to 3 days, 3 = once a week, to 4 = less often than once a week.

##### TikTok video posting

Following Scherr and Wang (2021), we also captured whether individuals had ever recorded and posted a TikTok video themselves (coded as 1) or not (coded as 0).

### Data analysis

First, we used confirmatory factor analysis (CFA) to establish measurement models for essential constructs. This step was essential to make sure that subscale scores within the motives for TikTok use could be distinguished and reported independently. The root mean square error of approximation (RMSEA), the standardized root mean square residual (SRMR), the Tucker–Lewis index (TLI), and the comparative fit index (CFI) were among the fit statistics used to confirm the model fit and ensure that the measurement was accurate. In accordance with Iacobucci (2010), the model fit was deemed adequate if the following criteria were met: for the CFI and TLI, values close to or higher than 0.95 were preferred, but values as low as 0.90 were acceptable; the value for the RMSEA was preferred as low as possible, although value below 0.80 was regarded as acceptable. It should be stressed that these criteria cannot be interpreted as strict laws because they rely on complicated measuring models, variable treatment, and a large number of components (Marsh et al., 2004).

Second, we utilized the statistical program Mplus 8.3 (Muthén and Muthén, 2012) to categorize TikTok users into homogeneous profiles based on their motivations. As the number of anticipated profiles was unknown, we performed an exploratory analysis by comparing models for one to six profiles. We checked whether the Bayesian information criterion (BIC) and the Akaike information criterion (AIC) were applicable, and smaller BIC and AIC values indicated a better model fit (Akaike, 1974; Schwarz, 1978). Additionally, a significant value of p from the Lo–Mendell–Rubin likelihood ratio test (LMR-LRT) showed that the k profile model suited the data better than the k-1 profile model (Lo et al., 2001). The entropy was measured from 0 to 1, with greater than 0.70 indicating a good classification accuracy (Jung and Wickrama, 2008). Finally, in line with Marsh et al. (2009), we added another selection criterion that included the size of the profiles (profiles with less than 5% of the sample were not excellent) and their interpretability.

Third, multinomial logistic regression employing profile membership as the dependent variable, TikTok use (frequency, daily use time, and video posting) and background characteristics as potential predictor variables, was carried out. This analysis was done to explore the predictive effects of TikTok use (frequency, daily use time, and video posting) and background characteristics for the TikTok use motives profiles.

## Results

### Measurement models and descriptive statistics

Overall, the model for motives for TikTok use was acceptable (RMSEA = 0.089, CFI = 0.906, TLI = 0.889). We present the means, standard deviations, and correlations of all indicator variables in Table 2. The correlations suggest that the motivation-related variables (8–11) are closely related to each other. Furthermore, the correlations suggest that there is an association between the TikTok use variables (5–7) and the motivation variables, especially the latter three subscales (9–11). These results were consistent with the correlational results in Scherr and Wang (2021).

**Table 2:** Means, standard deviations for profile variables, and correlations among study variables.

	1	2	3	4	5	6	7	8	9	10	11
1. Age	/	−0.04	−0.319^**^	0.366^**^	0.05	−0.06	0.186^**^	0.159^**^	0.198^**^	0.03	0.07
2. Gender		/	−0.07	−0.162^**^	0.00	0.05	0.09	0.05	0.00	−0.01	0.01
3. Degree			/	0.07	0.04	−0.01	−0.288^**^	−0.279^**^	−0.193^**^	0.02	0.00
4. Monthly income				/	−0.03	−0.02	−0.08	−0.08	−0.03	−0.01	−0.05
5. Frequency					/	−0.540^**^	−0.173^**^	−0.10	−0.174^**^	−0.364^**^	−0.196^**^
6. Daily use time						/	0.153^**^	0.09	0.161^**^	0.282^**^	0.197^**^
7. Video posting							/	0.476^**^	0.310^**^	0.186^**^	0.128^*^
8. Socially Rewarding Self-Presentation								/	0.757^**^	0.583^**^	0.487^**^
9. Trendiness									/	0.641^**^	0.628^**^
10. Escapist addiction										/	0.718^**^
11. Novelty											/
*M*	27.74	/	/	/	/	/	/	3.15	3.34	3.59	3.69
*SD*	8.92	/	/	/	/	/	/	1.02	1.04	0.93	1.05

M, means; SD, standard deviation; gender (1 = male, 2 = female) and video posting (0 = no, 1 = yes) were binary variable; Degree and Monthly income were category variables. ∗*p* < 0.05; ∗∗*p* < 0.01.

### Motives for TikTok use profiles

To answer the first research question regarding the identification of TikTok user profiles, fit indices and criteria are used for the selection of the model with the optimal number of clusters (see Table 3). The four-profile solutions provided the best overall model fit to the data. The four-profile solution had the highest entropy value (0.91). Although the BIC and AIC for the four-profile solution were slightly higher (BIC = 3481.61, AIC = 3390.75) than that of the five- (3443.97, 3333.35) and six-profile solutions (3437.08, 3306.71), the four-profile solution was chosen upon examination of the LMR. The LMR tests indicated that the four-profile solution was an improvement over the three-profile solution (*p* = 0.025), but the five-profile solution was not an improvement over the four-profile solution (*p* = 0.760). Finally, the four-profile model also had an acceptable profile breakdown (each profile with over 10% of the sample).

**Table 3:** Fit indices for different models with the number of clusters ranging from 1 to 6.

Clusters	1-profile	2-profile	3-profile	4-profile	5-profile	6-profile
# of free parameters	8	13	26	**23**	28	33
BIC	4436.04	3943.25	6005.92	**3481.61**	3443.97	3437.08
AIC	4404.43	3891.90	5903.21	**3390.75**	3333.35	3306.71
Entropy	/	0.80	0.90	**0.91**	0.91	0.86
LMR-LRT	/	*p* = 0.025	*p* < 0.001	***p* = 0.025**	*p* = 0.760	*p* = 0.030
% sample/class						
1	384 (100%)	135 (35%)	42 (11%)	**42 (11%)**	41 (11%)	37 (10%)
2		249 (65%)	176 (46%)	**138 (36%)**	129 (34%)	74 (19%)
3			166 (43%)	**154 (40%)**	24 (6%)	80 (21%)
4				**50 (13%)**	38 (10%)	38 (10%)
5					151 (39%)	134 (35%)
6						21 (5%)

*N* = 384. Bold indicates the best fit.

**Figure 1:**
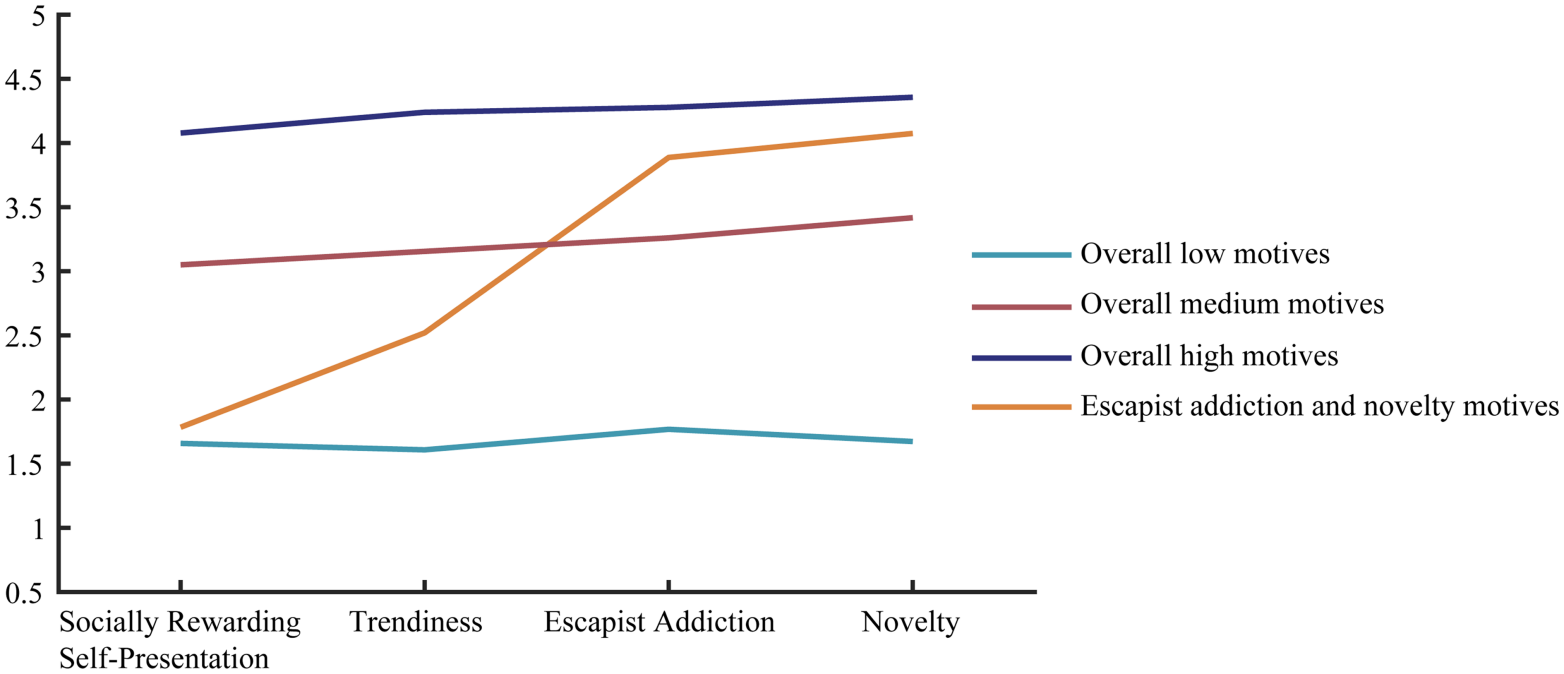
Profiles of motives for TikTok use.

The labeling of the profiles is based on the terminology of previous studies on motives for using Facebook profiles (e.g., Cristescu and Balog, 2018). Figure 1 shows the different profiles and their means for each TikTok use motive subscale. The first profile is called the “overall low motives profile” (*n* = 42), and users in this profile have the lowest scores for all subscales of motives for using TikTok. The second profile is called the “overall medium motives profile” (*n* = 138), and users in this profile are characterized by moderate scores on all subscales. The third profile is called the “overall high motives profile” (*n* = 154), namely the profile in which users score highest for all subscales of motives for using TikTok. The fourth profile is characterized by low scores on socially rewarding self-presentation and trendiness subscales but high scores on escapist addiction and novelty, so we called this profile the “escapist addiction and novelty motives profile” (*n* = 50).

The means and standard errors estimated for each of the subscale variables used in deriving the profiles are provided in Table 4. To further substantiate the significant differences in the means of these variables across profiles, a one-way MANOVA is used with all motivation-related variables as outcomes and the profile membership as the grouping variable. Results showed a significant effect of profile membership on the motives of TikTok users, Wilks’ lambda (Λ) = 0.05, *F*(12, 998) = 171.63, *p* < 0.001, η_p_^2^ = 0.628. Tukey’s HSD *post-hoc* tests suggest significant differences across all profiles in all motives for using TikTok subscales except for the difference between the first and fourth profiles in the socially rewarding self-presentation subscale (see Table 4).

**Table 4:** Tukey HSD *post hoc* comparisons among the four profiles.

	Overall low motives profile *n* = 42 (11%) *M* (*SE*)	Overall medium motives profile *n* = 138 (36%) *M* (*SE*)	Overall high motives profile *n* = 154 (40%) *M* (*SE*)	Escapist addiction and novelty motives profile *n* = 50 (13%) *M* (*SE*)
*Variables defining latent profiles*				
Socially rewarding self-presentation	1.67 (0.07) _a_	3.06 (0.04) _b_	4.09 (0.04) _c_	1.77 (0.06) _a_
Trendiness	1.61 (0.09) _a_	3.15 (0.05) _b_	4.25 (0.05) _c_	2.54 (0.08) _d_
Escapist addiction	1.78 (0.08) _a_	3.25 (0.04) _b_	4.28 (0.04) _c_	3.89 (0.07) _d_
Novelty	1.67 (0.10) _a_	3.42 (0.06) _b_	4.36 (0.05) _c_	4.08 (0.09) _d_

Means in the same row with different subscripts differ significantly at *p* < 0.05.

### Predicting TikTok use motives profile membership

Multinomial logistic regression is conducted with TikTok use (daily use time, frequency, and TikTok video posting) and background characteristics (i.e., age, gender, highest obtained degree, and monthly income) as possible predictors of profile membership. This analysis is conducted to explore the extent to which TikTok use and personal background characteristics of TikTok users predict their profile membership. The overall low motives profile is used as the reference category. First, the likelihood of membership in the overall medium motives profile is compared to the membership in the overall low motives profile. As shown in Table 5, positive significant effects are found for monthly income (*p* < 0.05; OR = 3.53) and high frequency (every day) (*p* < 0.05; OR = 3.48). More specifically, TikTok users with a monthly income lower than 5,000 were three times more likely to be in the overall medium motives group (class 2) vs. the overall low motives (class 1) than those who had a higher monthly income (OR = 3.53). TikTok users who used the app every day were three times more likely to be in the overall medium motives group (class 2) vs. the overall low motives (class 1) than those who used it less often than once a week (OR = 3.48).

**Table 5:** Multinomial logistic regression results predicting profile membership.

	Overall medium motives profile vs. Overall low motives profile			Overall high motives profile vs. Overall low motives profile			Escapist addiction and novelty motives profile vs. Overall low motives profile		
	B (SE)	OR	95% CI	B (SE)	OR	95% CI	B (SE)	OR	95% CI
Background characteristics									
Age	−0.02 (0.03)	0.98	[0.92,1.04]	0.00 (0.03)	1.00	[0.94,1.06]	0.04 (0.04)	1.04	[0.96,1.13]
Gender	0.16 (0.39)	1.17	[0.54,2.51]	0.14 (0.39)	1.15	[0.53,2.50]	0.50 (0.49)	1.66	[0.64,4.31]
Student status	0.93 (0.68)	2.54	[0.67,9.66]	1.22 (0.68)	3.40	[0.90,12.82]	0.48 (0.87)	1.62	[0.29,8.98]
Highest degree	−0.83 (0.49)	0.44	[0.17,1.14]	−0.51 (0.47)	0.60	[0.24,1.51]	−3.17 (1.25)*	0.04	[0.00,0.49]
Monthly income	1.26 (0.53)*	3.53	[1.25,9.96]	0.96 (0.51)	2.61	[0.96,7.12]	1.16 (0.77)	3.19	[0.71,14.35]
TikTok use									
Daily time (<10 min)	−0.52 (0.66)	0.59	[0.16,2.17]	−0.73 (0.65)	0.48	[0.13,1.73]	−0.37 (0.91)	0.69	[0.12,4.12]
Daily time (11–30 min)	−0.07 (0.64)	0.93	[0.27,3.25]	−0.50 (0.63)	0.61	[0.18,2.07]	−0.61 (0.76)	0.54	[0.12,2.40]
Daily time (31–60 min)	0.24 (0.67)	1.27	[0.34,4.68]	0.03 (0.65)	1.03	[0.29,3.72]	−1.00 (0.80)	0.37	[0.08,1.75]
Frequency (every day)	1.25 (0.58)*	3.48	[1.12,10.84]	1.65 (0.59)**	5.22	[1.64,16.55]	3.07 (0.84)**	21.53	[4.17,111.28]
Frequency (every 2–3 days)	−0.19 (0.55)	0.82	[0.28,2.42]	1.00 (0.54)	2.72	[0.94,7.87]	0.39 (0.89)	1.47	[0.26,8.36]
Frequency (once a week)	−0.19 (0.60)	0.82	[0.26,2.66]	0.70 (0.59)	2.01	[0.63,6.41]	−0.66 (1.20)	0.52	[0.05,5.41]
Video posting (never)	0.19 (0.40)	1.21	[0.55,2.68]	−1.04 (0.40)*	0.35	[0.16,0.78]	3.36 (0.85)**	28.69	[5.44,151.21]

∗*p* < 0.05; ∗∗*p* < 0.01; OR, odds ratio; CI, confidence interval. Reference category for gender is “female”; for student status is “Not student”; for highest obtained degree is “bachelor’s degree or higher”; for monthly income is “more than 5000”; for daily use time is “more than 1 h”; for frequency is “less often than once a week”; for video posting is “has ever posted a TikTok video.” Please unbold the values. It indicates significant results, which has also been indicated by * or **.

Second, comparison between the overall high motives profile and the overall low motives profile indicates that TikTok users who used the app every day were five times more likely to be in the overall high motives group (class 3) vs. the overall low motives (class 1) than those who used it less often than once a week (*p* < 0.01; OR = 5.22). Moreover, it shows that video posting has a negative significant effect (*p* < 0.05; OR = 0.35). TikTok users who had never posted a video on the app were 0.35 times less likely to be in the overall high motives group (class 3) vs. the overall low motives (class 1) than those who had ever posted a video.

Last, comparison between the escapist addiction and novelty motives profile and the overall low motives profile also indicates a positive effect of high frequency (every day) (*p* < 0.01; OR = 21.53) and a negative effect of video posting (*p* < 0.01; OR = 28.69). In addition, a negative effect of highest obtained degree was revealed (*p* < 0.05; OR = 0.04). TikTok users who did not obtain a bachelor’s degree were 0.04 times less likely to be in the escapist addiction and novelty motives group (class 4) vs. the overall low motives (class 1) than those who had at least a bachelor’s degree. No significant effects of any of the age, gender, student status, or daily use time on the profile membership were detected.

## Discussion

Due to the diversity of their personal experiences, TikTok users are heterogeneous. Our goal in this study was to explore profiles of TikTok users in terms of what motivates them to use it. We adopted latent profile analysis (LPA) to group participants into meaningful groups. We also conducted multivariate analysis of variance (MANOVA) and multinomial logistic regression to further explore the differences in TikTok use between the profiles and the extent to which users’ background characteristics and TikTok use (daily use time, frequency, and video posting) predict their profile membership. In this section, we also discuss limitations and suggestions for future study. Finally, we wrap up some useful implications for TikTok usage.

### TikTok use motives profiles

The results of LPA yielded the presence of four profiles, overall low motives, overall medium motives, overall high motives, and escapist addiction and novelty motives. Consistent with previous research on motivations for using social media (Cristescu and Balog, 2018), the current study revealed the former three profiles reflecting different levels of motivations: overall low, medium, and high. It is notable that the current study also yielded a fourth profile (namely the ‘escapist addiction and novelty motives profile’), which was characterized by high scores in escapist addiction and novelty, but low scores in socially rewarding self-presentation and trendiness. That is, participants in this group used TikTok mostly because they were unable to stop using it and addicted to new things on TikTok. The four profiles indicated that TikTok differed from other popular SNSs in motives for using, and gratifications derived from it.

The four profiles differed significantly in terms of the scores of almost all TikTok use motivation subscales. The only exception is that no difference was found between the overall low motives and the escapist addiction and novelty motives profiles in terms of the socially rewarding self-presentation subscale. Participants in the escapist addiction and novelty motives profile scored higher than the overall medium motives profile but lower than the overall high motives profile in both the escapist addiction and novelty subscales. Moreover, their scores on the trendiness subscale were somewhere in between overall low motives and medium motives profiles. These findings further indicated the uniqueness of the escapist addiction and novelty motives profile.

It is worth noting that the addictive feature of using TikTok was represented in both the overall high motives profile and the escapist addiction and novelty motives profile. Although participants in both groups scored high in the escapist addiction and novelty motives, they showed distinct characters in socially rewarding self-presentation and trendiness motives, resulting in two homogeneous subgroups of users. There is a lot of controversy and division regarding how social media affect young people’s mental health (Allen et al., 2014; Best et al., 2014). While some research highlighted the positive aspects of social media use, others showed the detrimental aspects. For instance, previous evidence showed that active use of social media was positively related to happiness (Marengo et al., 2021). It has also been shown that smartphone use was linked with clinical disorders (e.g., depression and addiction) (Stanković et al., 2021). Considering that the socially rewarding self-presentation motive was about social connection and active use (video posting), individuals in the escapist addiction and novelty motives profile might not benefit from the positive aspects of TikTok use. Therefore, tailed content recommendations should be provided based on different patterns of motives to achieve maximized gratifications and also maintain mental wellbeing.

### Predictive role of TikTok use and background characteristics

The focus on TikTok use motives is essential in adjusting the design and usability features of TikTok, to make it more adaptive to individual needs and gratifications. We conducted a multinomial logistic regression analysis to explore the extent to which TikTok use and personal background characteristics of TikTok users predict their profile membership. Results indicated that using TikTok every day relative to using it less often than once a week means a greater chance that users are members of the overall medium, high, and escapist addiction and novelty motives profile group instead of being a member of the overall low motives profile. The frequency of social media use was one of the most essential indicators in studies of youth social media use (Pempek et al., 2009). This finding in the current study was in line with previous research on the association between the frequency of social media use and perceived social media addiction (Allahverdi, 2022). In addition, no video posting means a lower chance as members of the overall high motives profile group and a greater chance as a member of the escapist addiction and novelty motives profile group instead of being a member of the overall low motives profile. This finding was in line with previous research showing that highly engaged TikTok users who often create and stream TikTok content, polish TikTok videos before posting, and engage or contribute TikTok content with others were motivated by social rewards (Meng and Leung, 2021). In contrast with Scherr and Wang (2021), results in the current study demonstrated no difference among users with a diversity of profiles regarding the daily use time of TikTok. Finally, in contrast with several other studies (e.g., Dhir et al., 2016; Noguti et al., 2019), the current study did not find any predictive effects related to age and gender. Compared to the overall low motives profile, the overall medium motives profile tended to have a low monthly income (lower than 5,000), and the escapist addiction and novelty motives profile tended to be highly educated. In general, the frequency of TikTok use and video posting are the most notable predictors of profile membership.

### Implications, limitations, and future research

The results of the current study offer several theoretical contributions and practical implications. First, this study makes theoretical contributions to the extant literature on social media use profiles (e.g., Scott et al., 2017; Lo Coco et al., 2018; Shensa et al., 2018) by exploring the number and types of latent profiles of TikTok use motives. Specifically, four profiles were revealed, namely overall low motives, overall medium motives, overall high motives, and escapist addiction and novelty motives profiles. Second, the study also showed that the frequency of TikTok use and video posting are the most notable predictors of profile membership and that four profiles differ significantly in terms of the scores of almost all TikTok use motives subscales except that no difference is found between the overall low motive and the escapist addiction and novelty motives profiles in terms of socially rewarding self-presentation. It is worth noting that the escapist addiction and novelty motives profile is unique, and it is crucial to pay special attention to individuals in the escapist addiction and novelty motives profile in terms of both gratifications and mental wellbeing. Together, the presence of distinct user profiles shows that TikTok users should not be assumed to be homogeneous. Understanding variations in the TikTok use motives could be useful in adjusting the design features of TikTok to both achieve maximized gratifications and maintain mental wellbeing by means of tailored content recommendations.

Although the present study offers important insights regarding diversity in TikTok use motives, several limitations must be addressed. First, our study is the first to explore the TikTok use motives profiles within a heterogeneous sample of users in China. Thus, the current findings could not extend to TikTok users in other countries. Future research could explore the cultural differences in further detail. Secondly, our relatively small sample prohibited us from examining variations among demographic groups. Our study recruited participants from a relatively large age range (17–58 years old), which might influence the predictive roles of age and gender in the TikTok use motives profiles. Therefore, future studies should incorporate a larger sample size of young users, given that the major user are under 30 years old (Doyle, 2022). Thirdly, we identified latent profiles based on four motives proposed by Scherr and Wang (2021), which did not include the motives for making money. As indicated in a previous study (Meng and Leung, 2021), making money also motivated TikTok usage. Future studies should consider more TikTok motives. Finally, we did not associate TikTok use motives profile with specific contents. For instance, the escapist addiction and novelty motives profile in the current study might link to addictive use and detrimental consequences (see Smith and Short, 2022). Future studies could tackle this limitation by incorporating the scenarios of TikTok use.

## Conclusion

User motives are crucial for the success of social media platforms. As TikTok is one of the most newly trendy social media, it is especially essential to investigate users’ motives to better address their needs and gratifications. Though a previous study has revealed four motives for TikTok use, person-centered research in this context is lacking. The present study fills this gap and expands the scientific literature on the TikTok use motives by adopting latent profile analysis (LPA). The results offer proof of the differences in TikTok use motives, by revealing four profiles, namely overall low, medium, high, and escapist addiction, and novelty motives profiles. Additionally, our findings of differences across profiles, particularly socially rewarding self-presentation and escapist addiction motives, are novel and notable. Furthermore, the current study helped to generate knowledge regarding the predictive effect of TikTok use frequency and active use (i.e., video posting). These results inform opportunities for enhancing user experience and gratifications of using TikTok based on user profiles.

## Data availability statement

The data generated during and/or analyzed during the current study are available from the corresponding authors on reasonable request.

## Ethics statement

Ethical review and approval were not required for the study on human participants in accordance with the local legislation and institutional requirements. Participants provided online informed consent before their enrollment in the study.

## Author contributions

LG: conceptualization, methodology, formal analysis, visualization, and writing—original draft. XG: visualization. YL: writing—review and editing. All authors contributed to the article and approved the submitted version.

## Funding

This research was supported by the Humanity and Social Science Youth Foundation of Ministry of Education of China (22YJCZH037) to LG.

## Conflict of interest

The authors declare that the research was conducted in the absence of any commercial or financial relationships that could be construed as a potential conflict of interest.

## Publisher’s note

All claims expressed in this article are solely those of the authors and do not necessarily represent those of their affiliated organizations, or those of the publisher, the editors and the reviewers. Any product that may be evaluated in this article, or claim that may be made by its manufacturer, is not guaranteed or endorsed by the publisher.

## Footnotes

^1^ https://www.wjx.cn/
